# Does adjuvant therapy improve survival in patients undergoing pancreaticoduodenectomy for distal cholangiocarcinoma? A systematic review, meta-analysis and meta-regression

**DOI:** 10.1007/s13304-025-02383-y

**Published:** 2025-09-01

**Authors:** G. Nappo, V. D’Ambra, C. Ricci, M. Pagnanelli, V. Rebasti, G. Alves, S. Crippa, L. Rimassa, R. Casadei, A. Zerbi

**Affiliations:** 1 Pancreatic Surgery Unit, IRCCS Humanitas Research Hospital, Rozzano, Italy; 2 Department of Biomedical Sciences, Humanitas University, Pieve Emanuele, Italy; 3 Pancreatic Surgery Unit, IRCCS Azienda Ospedaliero-Universitaria Di Bologna, Via Massarenti 9, 40138 Bologna, Italy; 4 Pancreatic Surgery Unit, Pancreas Translational and Clinical Research Centre, IRCCS San Raffaele Scientific Institute, Milan, Italy; 5 Medical Oncology and Hematology Unit, Humanitas Cancer Center, IRCCS Humanitas Research Hospital, Rozzano, Italy

**Keywords:** Distal cholangiocarcinoma, Adjuvant therapy, Pancreatoduodenectomy, Survival, Meta-analysis

## Abstract

The role of adjuvant therapy (AT) after pancreaticoduodenectomy (PD) for distal cholangiocarcinoma (DC) remains controversial. This systematic review and meta-analysis aimed to evaluate the impact of AT on overall survival (OS) and disease-free survival (DFS) in patients with resected DC. A systematic review was conducted following PRISMA guidelines (PROSPERO: CRD42024561780). PubMed, Scopus, Web of Science, and Cochrane databases were searched for studies published between January 2000 and August 2023. Eligible studies included patients who underwent PD for DC, comparing AT to follow-up (FUP) alone. Primary endpoints were OS and DFS, analyzed using hazard ratios (HR) with 95% confidence intervals (CI). Meta-regression explored potential sources of heterogeneity. The analysis included 22 studies with 7078 patients (3860 FUP group, 3218 AT group): of the studies, only 3 (13.6%) were RCTs (even if none of them was specifically designed for DC). AT significantly improved OS (HR 0.80; 95% CI 0.73–0.89; *p* < 0.001) and DFS (HR 0.84; 95% CI 0.74–0.96; *p* = 0.023) compared to FUP. Subgroup analysis showed a greater benefit for chemo-radiotherapy (OS HR 0.81; 95% CI 0.72–0.91) over chemotherapy alone (OS HR 0.78; 95% CI 0.64–0.94). High heterogeneity (*I*^2^ = 98%) was identified among the included studies. This meta-analysis demonstrates that AT is associated with improved survival outcomes in patients undergoing PD for DC. However, the significant limitations of the existing literature, particularly the lack of RCTs designed specifically for DC, necessitate caution, especially on which type of chemotherapy should be adopted.

**Supplementary Information:**

The online version contains supplementary material available at 10.1007/s13304-025-02383-y.

## Introduction

Distal Cholangiocarcinoma (DC) is a malignancy of the biliary tree that arises distal to the cystic duct up to the entrance of the common bile duct into the wall of the duodenum. DC represents approximately 30% of all cholangiocarcinoma subtypes [1, 2], with an incidence of 0.3–0.5 per 100,000 inhabitants in Western countries [3, 4]. The only potential curative treatment for DC is surgical resection, typically consisting of PancreaticoDuodenectomy (PD), combined with lymphadenectomy. However, the prognosis remains poor, as many patients present with advanced local or distant disease at diagnosis [5]: even for patients undergoing surgical resection, the 5‐year Overall Survival (OS) rate ranges between 20 and 66%, largely due to frequent disease recurrence [6–10].

The role of Adjuvant Therapy (AT) after resection for DC remains uncertain and is a topic of ongoing debate [11, 12]. Two major randomized controlled trials (RCTs), PRODIGE-12 and BCT, failed to demonstrate a survival benefit with AT in biliary tract cancer [13, 14]. However, the BILCAP Trial [11], in a prespecified per-protocol analysis, showed improved survival with adjuvant capecitabine; these data were, subsequently, confirmed when longer follow-up was available [15]. Similar results were found in another eastern RCT (ASCOT), on which a significant improvement in survival was found after administration of adjuvant S-1 after resection for biliary tract cancer [16]. Consequently, current international guidelines recommended 6 months of adjuvant capecitabine for patients with resected biliary tract cancer [17–19]. Nonetheless, a systemic review and meta-analysis of three RCTs focusing on biliary tract cancer showed no significant effect on OS [20]. The heterogeneity of the published studies, which included various subtypes of cholangiocarcinoma (intrahepatic, perihilar, gallbladder and DC), makes interpreting these findings challenging [11, 13, 14, 21, 22]. More recent analyses using Propensity Score-Matching (PSM) have provided conflicting evidence regarding the survival benefits of postoperative AT for DC [23, 24]. To date, no systematic review and meta-analyses has focused exclusively on the impact of AT in patients who have undergone curative‐intent resection for DC.

Given this gap in the literature, we conducted a systematic review and meta-analysis to evaluate the prognostic impact of AT in patients who underwent resection for DC.

## Methods

The systematic review was conducted according to the PRISMA guidelines [25]. This protocol was registered with PROSPERO 2024 (registration number CRD42024561780).

### Search strategy

A comprehensive search strategy was developed using the PICO methodology [26]:

- Population: patients who underwent resection for DC
- Intervention: administration of AT post- resection (ADJ group);
- Control: patients who underwent follow-up after resection (FUP group);
- Outcomes: OS and Disease-Free Survival (DFS).

A systematic literature search was performed using PubMed/Medline, Scopus, Web of Science and Chochrane databases. Eligible studies published between January 2000 and August 2023 were included. The search terms used were: “(“Cholangiocarcinoma” [Mesh] OR “Extrahepatic Cholangiocarcinoma” [tw] OR “Distal Cholangiocarcinoma” [tw]) AND (“Chemotherapy, adjuvant” [Mesh] OR “Adjuvant chemotherapy” [tw] OR “Adjuvant drug therapy” [tw] OR “radiation therapy” [tw] OR “radiotherapy” [tw])”. The SR accelerator tool was used to translate the search string for Scopus, Cochrane and Web of Science [27]. Only studies published in English were included. Additionally, references of all included studies and other relevant articles were hand-searched to ensure comprehensiveness.

### Selection criteria

Two independent investigators (DV, PM) screened all records identified through the literature search. Predefined inclusion and exclusion criteria were applied. Case-report, meta-analyses, reviews, editorials, and expert opinions were excluded. If abstracts appeared relevant, full articles were assessed for eligibility. Only studies reporting survival data (OS and/or DFS) for both ADJ and FUP groups after resection for DC were considered. Studies where survival data for DC could not be separated from the other subtypes of cholangiocarcinoma (intrahepatic, perihilar or gallbladder carcinoma) were excluded. In case of overlapping cohorts, the most recent or relevant publication was selected.

### Outcomes measures and data extraction

The primary outcomes were to evaluated differences in OS and DFS between ADJ and FUP groups. Two independent authors (DV and PM) extracted data based on a pre-specified protocol. Any disagreements were resolved by a third reviewer (NG). Collected data included: author, year, study design, country, adjuvant chemotherapy regimen, radiotherapy, male sex, age, TNM stage, grading, radical resection R0, ASA score, perineural invasion (PNI), lymph-vascular invasion (LVI), survival data (OS and/or DFS).

### Methodological quality

The methodological quality of the included retrospective studies was assessed using the validated Methodological Index for Non-Randomized Studies (MINORS) [28].

### Statistical analysis

Survival data for ADJ and FUP groups were primarily reported as Hazard Ratio (HR); when HR was not provided, it was obtained from Kaplan–Meyer curve analysis according to the Parmar method [29, 30].

The Mantel–Haenszel random effects model was used to calculate the effect size [31]. The results were reported as hazard ratios (HR) with 95% confidence intervals (95 CI). A two-tailed *p* value < 0.05 indicated a non-negligible effect.

The risk of bias across included studies was tested, measuring both the "between-study heterogeneity" and publication bias. The heterogeneity between studies was tested using the *I*^2^ [32]. The heterogeneity was interpreted as follows: If *I*^2^ was < 50%, the risk of "between-study" heterogeneity was considered low–moderate, and if *I*^2^ was ≥ 50%, it was judged high. The meta-regression analysis was carried out if the heterogeneity was judged high [33, 34]. Among the included papers, baseline data were found to be quite heterogeneous. For this reason, a Random Effect Model was applied for the analysis. In the first step, we calculated the distribution of confounding covariates among each arm, reporting the results as Risk Ratio (RR), Mean Difference (MD), or percentage. In the second step, *β* coefficient with standard error (SE) and *R*^2^ was reported. The beta coefficient ± SE was related to the change in the HR of the event: a positive beta coefficient means that the covariate increased rate generates a positive HR modification. The *R*^2^ measured the quote, in percentage, of the heterogeneity explained by the variable. A two-tailed *p* value < 0.05 was judged significant. The *p* values were also recalculated using Monte Carlo permutation to obtain solid results. Publication bias was assessed with a funnel plot and Egger and Begg tests, and a *p*-value < 0.05 indicated a non-negligible "small-study effect” [35, 36].

This meta-analysis was performed using the statistic software R with dedicated packages.

## Results

**Fig. 1:**
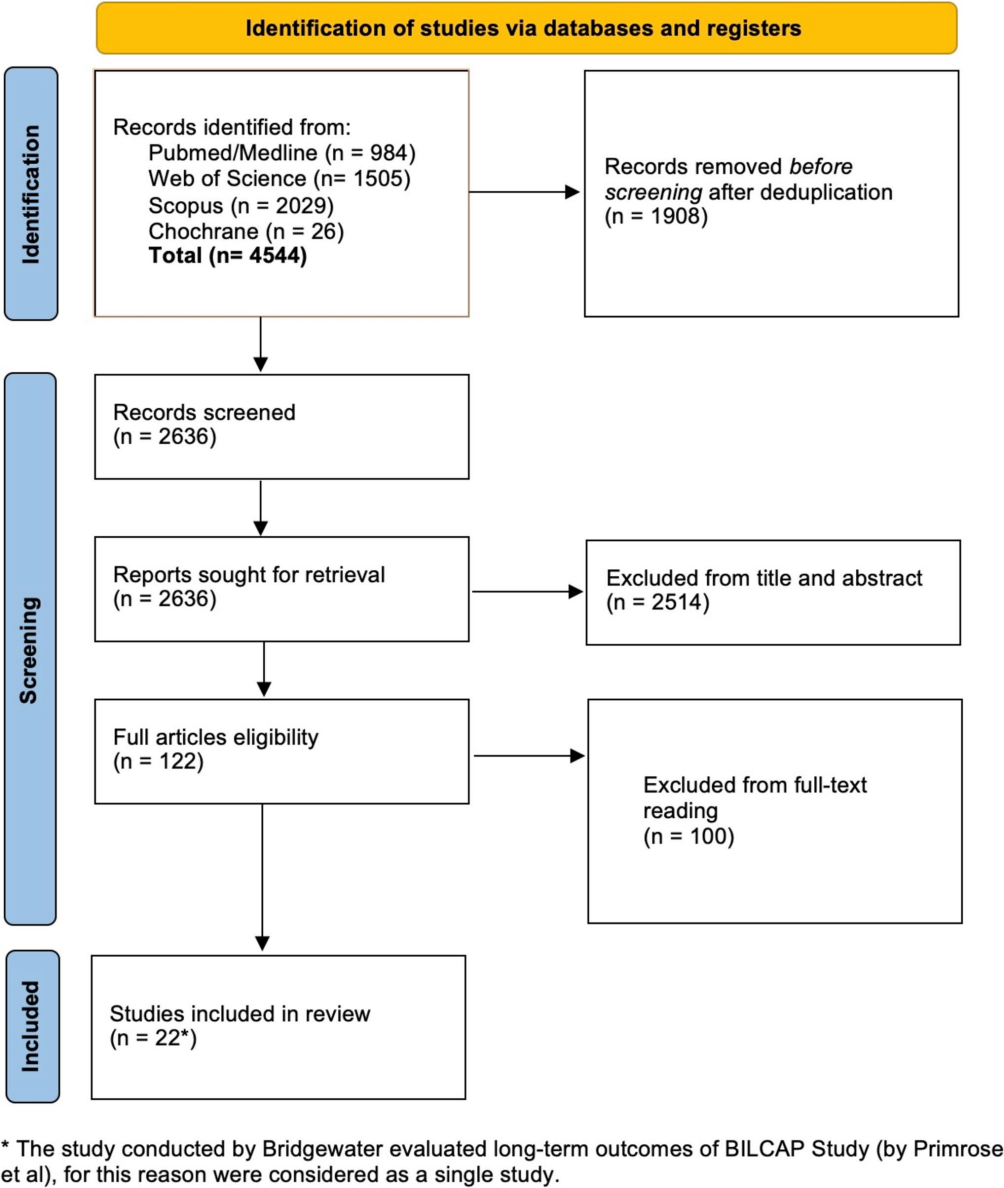
PRISMA flow-chart of the study search and screening process

The selection process is outlined in Fig. 1. The search yielded 4544 records from Scopus, Pubmed/Medline, Web of Science and Cochrane databases. After screening, 122 records were fully reviewed, and 22 studies were included in the final analysis [11, 14–16, 37–52], comprising a total of 7078 patients (3860 in FUP group and 3218 in ADJ group). The article by Bridgewater et al. [15] was considered together with the long-term follow-up data from the BILCAP study (Primrose et al. [11]), treating them as a single study for this analysis. The characteristics of the included studies are summarized in Table 1. Fifteen studies (68.2%) were conducted in Western countries [11, 15, 23, 24, 37, 38, 40–42, 44, 47–52]. Of the studies, 18 (81.8%) were retrospective [37–39, 41–46, 48–52] (including 4 that used the PSM PSM [23, 24, 40, 47]), while only 3 (13.6%) were RCTs [11, 14–16]. One study involved radiotherapy alone as adjuvant treatment [37], while the rest evaluated chemotherapy ± radiotherapy. Information on nodal (N) and margin status (R) in both the surgery-alone and adjuvant therapy groups was available only in a limited number of included studies. Among the studies that reported detailed information on the adjuvant regimen administered, gemcitabine was the most frequently used agent (355 patients), followed by 5-fluorouracil (276 patients), capecitabine (114 patients), and platinum-based combinations (44 patients). Other less common regimens were reported in 29 patients. These data are summarized in Table 2.

**Table 1:** Characteristics of included studies (n = 22)

Author	Year	Country	Study Design	MINORS	No	Surgery alone	Surgery + Adjuvant	Chemotherapy/Radiotherapy regimen evaluated	N0 S^	N0 A^&^	R0 S^	R0 A^&^
Heron et al. [37]	2003	USA	Retrospective	10	30	17	13	Radiotherapy	16	11	–	–
Hughes et al. [38]	2007	USA	Retrospective	7	64	30	34	Chemotherapy ± Radiotherapy	19	6	28	25
Kim et al. [39]	2016	Korea	Retrospective	14	158	102	56	Chemotherapy ± Radiotherapy	77	34	102	56
Ecker et al. [40]	2017	USA	Retrospective with PSM	13	790	366	424	Chemotherapy ± Radiotherapy	–	–	–	–
Ethun et al. [41]	2017	USA	Retrospective	8	224	96	128	Chemotherapy ± Radiotherapy	–	–	–	–
Bergeat et al. [24]	2018	France	Retrospective with PSM	18	96	48	48	Chemotherapy ± Radiotherapy	14	7	6	12
Ebata et al. [14]	2018	Japan	RCT	–	123	57	66	Gemcitabine	–	–	–	–
Hester et al. [23]	2018	USA	Retrospective with PSM	15	1000	500	500	Chemotherapy ± Radiotherapy	228	238	379	378
Bolm et al. [42]	2019	Germany	Retrospective	10	118	88	30	Chemotherapy and/or Radiotherapy	–	–	–	–
Hoshimoto et al. [43]	2019	Japan	Retrospective	8	53	45	8	Chemotherapy	–	–	–	–
Strijker et al. [44]	2019	Netherlands	Retrospective	8	620	574	46	Chemotherapy ± Radiotherapy	–	–	–	–
Zhou et al. [45]	2020	China	Retrospective	12	124	96	28	Chemotherapy	67	13	96	28
Chang et al. [46]	2021	Korea	Retrospective	10	294	146	148	Chemotherapy and Radiotherapy	–	–	–	–
Kamarajah et al. [47]	2021	USA	Retrospective with PSM	11	1509	429	1080	Chemotherapy	–	–	–	–
Bridgewater [15]/Primrose [11]*	2022	UK	RCT	–	156	80	76	Capecitabine	–	–	–	–
De Jong et al. [48]	2022	Netherlands	Retrospective	12	625	557	68	Chemotherapy ± Radiotherapy	–	–	–	–
Nakachi et al. [16]	2023	Japan	RCT	–	158	80	78	S–1	–	–	–	–
Hou et al. [49]	2023	USA	Retrospective	12	245	95	150	Chemotherapy ± Radiotherapy	66	58	76	115
Macfie et al. [50]	2023	USA	Retrospective	9	41	21	20	Chemotherapy ± Radiotherapy	–	–	–	–
Tjaiden et al. [51]	2023	Germany	Retrospective	13	172	95	77	Chemotherapy ± Radiotherapy	44	20	63	37
Uijterwijk et al. [52]	2023	Italy	Retrospective	14	478	338	140	Chemotherapy	–	–	–	–

MINORS = Methodological Index for Non-Randomized Studies; No = sample size; PSM = Propensity Score Matching; RCT = Randomized Controlled Trial; * = the study conducted by Bridgewater et al. evaluated long-term outcomes of BILCAP Study (by Primrose et al.), for this reason were considered as a single study; ^ = Surgery alone; & = Surgery and Adjuvant

**Table 2:** Type of adjuvant chemotherapeutic regimens

Study	Platinum (N.)	Fluoropyrimidine (N.)	Gemcitabine (N.)	Other (N.)
Heron et al. [37]	0	0	0	0
Hughes et al. [38]	2	32	0	0
Kim et al. [39]	0	30	4	13
Ecker et al. [40]	N.A	N.A	N.A	N.A
Ethun et al. [41]	20	N.A	79	N.A
Bergeat et al. [24]	N.A	N.A	N.A	N.A
Ebata et al. [14]	0	0	66	0
Hester et al. [23]	N.A	N.A	N.A	N.A
Bolm et al. [42]	N.A	N.A	N.A	N.A
Hoshimoto et al. [43]	N.A	N.A	4	N.A
Strijker et al. [44]	N.A	N.A	N.A	N.A
Zhou et al. [45]	N.A	N.A	N.A	N.A
Chang et al. [46]	N.A	139	3	N.A
Kamarajah et al	N.A	N.A	N.A	N.A
Bridgewater [15]/Primrose [11] et al	0	76	0	0
De Jong et al. [48]	N.A	N.A	N.A	N.A
Nakachi et al. [16]	N.A	N.A	N.A	N.A
Hou et al. [49]	N.A	N.A	64	N.A
Macfie et al. [50]	N.A	N.A	N.A	N.A
Tjaden et al. [51]	N.A	N.A	68	N.A
Uijterwijk et al. [52]	22	35	67	16
**TOTAL**	**44**	**312**	**355**	**29**

NA: not assessed

### Methodological quality

The median MINORS score across the retrospective studies was 12 (7–18). Detailed MINORS scores for each study are presented in Supplementary materials, Fig. 1.

### Primary endpoint

Twenty studies reported data about OS [11, 14–16, 23, 24, 37–49, 51, 52], while 12 reported data about DFS [11, 14–16, 24, 39, 43, 45, 46, 49–52].

**Fig. 2:**
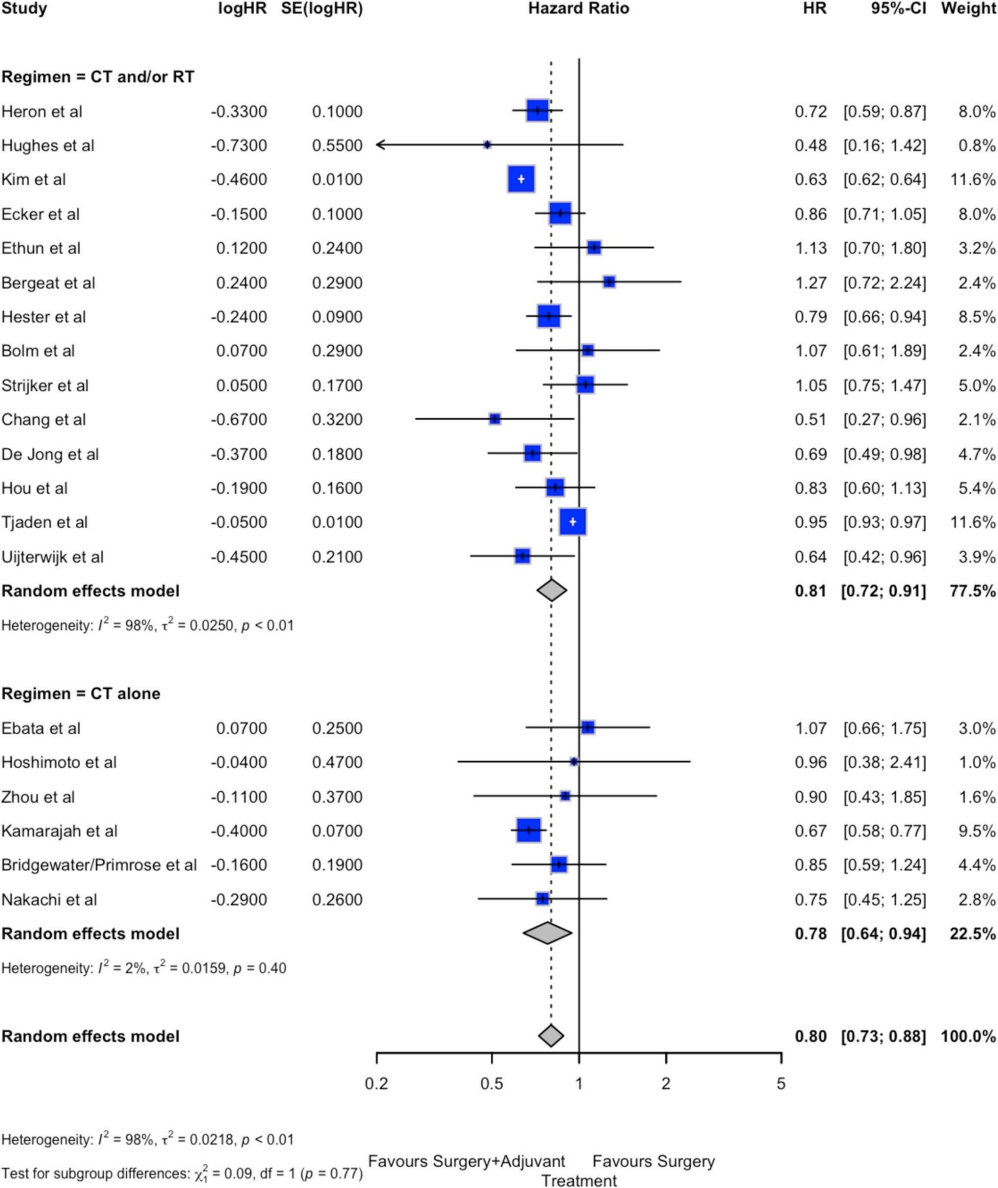

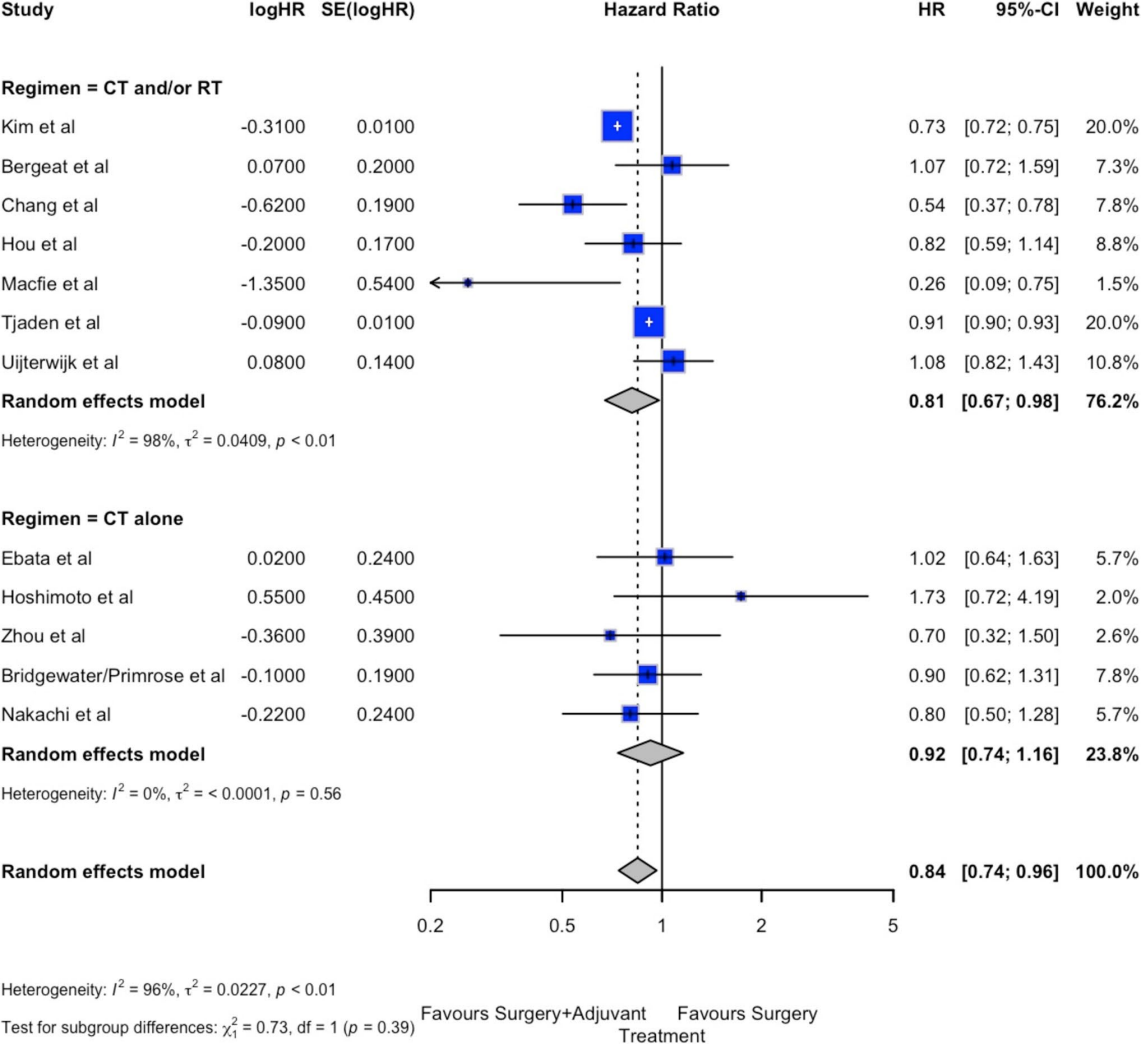
Forest plots of primary and secondary endpoints. A: overall survival. B: disease free survival

The forest plot of OS is shown in Fig. 2A. OS was significatively improved in the ADJ group compared to the FUP group (HR 0.80; 95%CI 0.73–0.89, *p* < 0.001). Similar results were observed for both the “adjuvant chemotherapy alone” or “chemotherapy and/or radiotherapy” subgroups: in both cases, OS was significantly longer in ADJ (HR 0.78; 95%CI 0.64–0.94 and HR 0.81; 95%CI 0.72–0.91, respectively). Despite the significant effect, heterogeneity was high (*I*^2^ = 98%), while the small-study effect was negligible (Egger *p* = 0.891, Begg *p* = 0.770, supplementary material Table 1 and Fig. 2A).

The forest plot for DFS is shown in Fig. 2B. The DFS in the ADJ group was significantly better than in the FUP group (HR 0.84; 95%CI 0.74–0.96, *p* = 0.023). For the “adjuvant chemotherapy alone” subgroup, the overall effect was similar between ADJ and FUP groups (HR 0.92; 95%CI 0.74–1.16); however, for the “chemotherapy and/or radiotherapy” subgroup, the DFS was significantly better in the ADJ group (HR 0.81; 95%CI 0.67–0.98). Heterogeneity for DFS was also high (*I*^2^ = 96%), while the small-study effect remained negligible (Egger *p* = 0.927, Begg *p* = 0.534, supplementary material Table 1 and Fig. 2B).

### Meta-regression

Due to the high heterogeneity observed in both primary and secondary endpoints, a meta-regression analysis was conducted to explore potential factors contributing to those variations. The univariate meta-regression analysis for OS and DFS are detailed in Tables 3 and 4, respectively.

**Table 3:** Univariate meta-regression analysis for primary endpoint (OS)

Covariates	Number of studies	Beta coefficient (SE)	Adjusted R^2^ (%)	P-value	P-value after Monte Carlo Permutation	Residue *I*^2^ (%)
Year	20	0.01 (0.01)	0,4	0.458	0.519	73.0
Study design	20	0.10 (0.17)	0	0.532	0.605	93.2
High Quality (PSM + RCT)	20	0.04 (0.11)	0	0.736	0.752	93.6
MINORS	17	− 0.01 (0.02)	0	0.801	0.809	91.1
East/West	20	0.16 (0.11)	25.1	0.138	0.203	57.7
Male sex (RR)	6	− 0.62 (0.18)	99.9	< *0.001*	0.111	0
Age (MD)	5	− 0.02 (0.02)	13.3	0.437	0.625	49.6
ASA (RR)	3	1.47 (1.10)	100	0.184	0.167	0
N0 (RR)	8	0.37 (0.19)	39.1	0.052	0.165	69.8
R0 (RR)	7	− 0.18 (0.46)	0	0.693	0.742	80.6
T1-T2 Stage (RR)	5	0.21 (0.43)	0	0.628	0.667	96.5
G1-G2 (RR)	6	− 0.08 (0.76)	0	0.914	0.914	55.0
PNI (RR)	4	− 0.10 (0.38)	0	0.793	0.833	0
LVI (RR)	3	− 0.34 (1.26)	0	0.788	1.000	0
Radiotherapy rate	12	− 0.27 (0.17)	7.8	0.113	0.220	79.8
Adjuvant platinum rate	8	0.68 (1.31)	0	0.604	0.655	54.7
Adjuvant fluoropyrimidine rate	8	− 0.10 (0.19)	0	0.591	0.745	31.6
Adjuvant gemcitabine rate	12	0.44 (0.10)	82.0	< *0.001*	*0.041*	19.6

SE = standard error; R^2^ = relative reduction in between-study variance, the value indicates the proportion of between-study variance explained by covariate; Residue *I*^2^ = Residue heterogeneity; MD = mean difference; RR = risk ratio; PSM = Propensity Score Matching; RCT = Randomized Controlled Trial; MINORS = Methodological Index for Non-Randomized Studies; R0 = Radical resection with negative margins; PNI = Perineural Invasion; LVI = Lymph-Vascular Invasion; 5-FU = 5-Fluorouracil

**Table 4:** Univariate meta-regression analysis for secondary endpoint (DFS)

Covariates	Number of studies	Beta coefficient (SE)	Adjusted R^2^ (%)	P-value	P-value after Monte Carlo Permutation	Residue * I*^2^ (%)
Year	12	0.01 (0.03)	0	0.794	0.813	52.2
Study design	12	0.09 (0.17)	0	0.627	0.650	96.5
High Quality (PSM + RCT)	12	0.16 (0.16)	0	0.351	0.303	96.1
MINORS	9	0.05 (0.04)	25.6	0.144	0.247	89.8
East/West	12	0.22 (0.01)	99.9	** < *0.001***	0.097	0
Male sex (RR)	4	− 0.78 (0.50)	100.0	0.119	0.292	0
Age (MD)	4	− 0.01 (0.03)	0	0.956	0.958	45.7
ASA (RR)	3	0.91 (0.90)	0	0.307	0.167	0
N0 (RR)	5	0.41 (0.03)	100.0	** < *0.001***	0.083	0
R0 (RR)	5	− 0.07 (0.33)	0	0.835	0.883	69.3
T1-T2 Stage (RR)	4	1.29 (0.08)	100.0	** < *0.001***	0.375	0
G1-G2 (RR)	3	− 0.83 (1.83)	0	0.649	0.833	0
PNI (RR)	4	− 0.24 (0.39)	0	0.544	0.417	0
LVI (RR)	3	0.41 (1.33)	0	0.757	1.000	0
Radiotherapy rate	8	− 0.52 (0.03)	100.0	** < *0.001***	***0.026***	0
Adjuvant platinum rate	5	2.02 (1.16)	63.7	0.082	0.400	26.7
Adjuvant fluoropyrimidine rate	6	− 0.38 (0.27)	27.2	0.157	0.261	61.5
Adjuvant gemcitabine rate	9	0.30 (0.14)	59.7	***0.031***	0.159	29.8

SE = standard error; R^2^ = relative reduction in between-study variance, the value indicates the proportion of between-study variance explained by covariate; Residue *I*^2^ = Residue heterogeneity; MD = mean difference; RR = risk ratio; PSM = Propensity Score Matching; RCT = Randomized Controlled Trial; MINORS = Methodological Index for Non-Randomized Studies; R0 = Radical resection with negative margins; PNI = Perineural Invasion; LVI = Lymph-Vascular Invasion; 5-FU = 5-Fluorouracil

For OS, meta-regression suggests that male sex and the use of adjuvant gemcitabine may have an impact on OS; however, only the use of gemcitabine still maintains its significance after correction using 5,000 Montecarlo permutation (*p* = 0.041). Interestingly, adjuvant fluoropyrimidine regimens did not show any significant effect on OS, both before and after corrections (*p* = 0.591 and 0.745); so, the fluoropyrimidine administration did not account for the observed between-study variability. Margin and nodal status did not significantly influence the association between adjuvant therapy and OS, suggesting that the observed benefit is not solely attributable to high-risk pathological subgroups. Other covariates, such as T1-2 stage, G1-2 differentiation, PNI, LVI, radiotherapy and adjuvant platinum administration, did not explain the high heterogeneity for OS, even after corrections via Monte Carlo permutation. This suggests that these have minimal influence on OS in the context of this meta-analysis.

Regarding DFS, meta-regression identified radiotherapy rate as the most statistically significantly consistent factor explaining the variance in outcomes after correction via Montecarlo permutation (*p* = 0.026). Margin and nodal status did not significantly influence the association between adjuvant therapy and DFS, suggesting that the observed benefit is not solely attributable to high-risk pathological subgroups. All other covariates were not significant after corrections.

## Discussion

The role of AT after resection for DC is still a matter of debate: this meta-analysis is the first to specifically evaluate the prognostic role of AT in the subset of patients who underwent resection for DC. We showed that AT significantly improves DFS (HR 0.84; 95%CI 0.74–0.96, *p* = 0.023) and OS (HR 0.80; 95%CI 0.73–0.89, *p* < 0.001).

However, these results must be carefully evaluated. It is important to consider the quality of the available literature, as only three of the included studies are RCTs [11, 14–16]. The retrospective design of most studies on this topic presents limitations, especially in controlling for confounding variables. Additionally, the MINORS scores range from 7 to 18, indicating a wide range of methodological quality, which complicates results’ interpretations. The studies vary significantly in sample sizes, with some cohorts exceeding 1000 patients [23, 47] and others comprising fewer than 50 [37, 50]; this variation in sample size contributes to the high heterogeneity observed in the meta-analysis outcomes. It is also noteworthy that none of the included studies clearly reported the criteria used to assign patients to the adjuvant therapy group. This lack of standardization limits the interpretation of treatment effect and reflects a common weakness of retrospective literature. Even in the three phase III randomized trials included (BILCAP, BCAT, PRODIGE-12) [11–13], while standardized eligibility criteria such as ECOG performance, timing post-surgery, and organ function were clearly defined, none reported clinical criteria used to decide administration of adjuvant therapy versus observation, such as postoperative complications or physician/patient preference. This limitation further hampers interpretation of treatment effects in real-world settings.

Another consideration is that the 3 RCTs [11, 14–16] were designed not specifically for DC: BCAT and ASCOT trials included patients with histologically proven extrahepatic bile duct cancer, including Klatskin tumors [14, 16]; BILCAP trial included all cases of cholangiocarcinoma, independently from the location (intrahepatic, perihilar, gallbladder or DC) [11, 15]. Consequently, the reported data for DC of these 3 RCTs were extrapolated from the entire cohort. This aspect raises important questions: should DC be considered similar to intrahepatic and perihilar cholangiocarcinoma, or should it be regarded as a different entity? When evaluating the efficacy of AT after resection for DC, is it sufficient and reliable to use results extrapolated from RCTs designed for all types of cholangiocarcinoma? The surgical approach, after all, differs greatly by tumor location (major or minor hepatectomy for intrahepatic and perihilar carcinomas, PD for DC). Moreover, the post-operative course also differs markedly, as patients undergoing PD often experience more severe complications, that may affect the administration of AT [53]. None of the included studies reported how many patients were clinically eligible for adjuvant therapy but ultimately did not receive it due to postoperative complications or impaired recovery. This represents a major limitation, as the tolerability and feasibility of adjuvant treatment after PD are key factors in real-world decision-making. It is also important to note that the primary aim of this meta-analysis was not to quantify the proportion of patients receiving adjuvant therapy after PD, but rather to assess its effect on long-term oncologic outcomes. To obtain reliable data on the real-world rate of AT administration after PD for DC, a prospective, multicenter observational study would be necessary. However, to date, no such prospective data have been published in the literature. Furthermore, can we confidently assert that perihilar or intrahepatic tumor exhibit the same biological behavior as DC? For this reason, in our opinion, DC should be regarded as a distinct entity and future RCTs on the role of AT after resection should be specifically designed for DC.

Another crucial consideration is the choice of adjuvant approach after resection for DC, whether to opt for chemotherapy alone or chemotherapy combined with radiotherapy. The majority of included studies adopted chemo-radiotherapy as the preferred strategy [23, 24, 38–42, 44, 46, 48–51]. In our meta-analysis, we separately analyzed the outcomes of chemo-radiotherapy and chemotherapy alone compared to FUP in terms of both DFS and OS. Our results suggest that both approaches of AT provide a survival benefit when compared to FUP; however, there appears to be a more substantial improvement in DFS with chemo-radiotherapy. Specifically, when considering chemotherapy alone, the overall effect was similar in ADJ group and in FUP group (HR 0.92; 95%CI 0.74–1.16); on the other hand, when analyzing chemo-radiotherapy, the overall effect was significatively better in ADJ group than FUP group (HR 0.81; 95%CI 0.67–0.98). This suggests that the addition of radiotherapy might play an important role in improving DFS by providing better local control and reducing recurrence rates. The meta-regression analysis further supports this conclusion, showing a significant positive effect of radiotherapy on DFS (*p* = 0.026). For OS, both chemotherapy alone and chemo-radiotherapy were associated with a statistically significant survival benefit compared to FUP (HR 0.78; 95%CI 0.64–0.94 and HR 0.81; 95%CI 0.72–0.91, respectively). This indicates that, regardless of whether radiotherapy is included, AT provides a significant improvement in survival for patients with resected DC. Moreover, none of the included studies reported survival outcomes stratified by resection margin or nodal status, and only a minority provided these variables at all. It was also not possible to determine whether radiotherapy was selectively administered in patients with R1 resections, due to incomplete reporting. It is important to note that current guidelines consider the radiotherapy only a possibility, but not a standard during AT for cholangiocarcinoma. In addition, no studies have directly compared chemoradiotherapy to chemotherapy alone as an adjuvant treatment for DC. Future RCTs designed specifically for DC are needed to directly compare these adjuvant strategies and clarify the optimal approach.

Determining the optimal chemotherapeutic regimen to be adopted remains challenging. While not a primary aim of this meta-analysis, we conducted a review of the chemotherapeutic agents reported in the included studies.. Unfortunately, most studies, particularly retrospective ones, did not provide detailed information on the chemotherapeutic strategies employed. For the 818 cases with available data, gemcitabine was the most commonly used agent (355 cases, 43.40%), followed by fluoropyrimidine-based regimens, such as 5-FU (276 cases, 33.7%) and capecitabine (114 cases, 13.49%). Interestingly, the three available RCTs adopted different regimens: BCAT study used gemcitabine [14], while the BILCAP [11, 15] and ASCOT [16] trials evaluated fluoropyrimidine-based chemotherapy (capecitabine and S-1, respectively). Based on the positive results from the BILCAP study, international guidelines currently recommend six months of adjuvant capecitabine following resection of biliary tract cancers [17–19]. It is noteworthy that none of the studies published after the BILCAP trial (except for the ASCOT one) adopted capecitabine; all instead evaluated gemcitabine-based therapies: this data is likely due to the retrospective nature of these studies, which included older patient cohorts operated before the BILCAP trial results were available. This further highlights the lack of consensus regarding the optimal adjuvant regimen for BTC and reinforces the need for disease-specific trials focusing on DC.

We performed a meta-regression to explore the potential prognostic impact of different chemotherapeutic agents: after correction for confounding variables, neither gemcitabine nor fluoropyrimidine-based chemotherapy (5-FU or capecitabine) demonstrated a significant survival benefit in terms of OS or DFS. This suggests that, in the specific setting of DC, no single chemotherapeutic agent has clearly demonstrated superiority as AT Moreover, the only published RCT comparing two adjuvant chemotherapy regimens after resection for DC in node positive patients (gemcitabine + cisplatin vs capecitabine) failed to demonstrate a significant difference in survival [54]. Therefore, despite the current guidelines recommending capecitabine as the standard of care following resection for biliary tract cancer, our findings and the available literature highlight the need for further research specifically focused on DC to determine the most effective adjuvant chemotherapeutic regimen in this setting. The only other planned RCT (ATICCA-1) comparing two adjuvant chemotherapeutic regimens (gemcitabine + cisplatin vs capecitabine) is ongoing and the results will be available soon [55]; notably, this study is designed to include all cases of cholangiocarcinoma, regardless of tumor location, as well as cases of gallbladder cancer.

The strength of this meta-analysis lies in its comprehensive evaluation of the available evidence on the role of AT after resection for DC. By aggregating multiple studies, it has strengthened the statistical power and highlighted the potential survival benefits associated with AT. However, this metanalysis has some limitations, including variability in study quality, potential publication bias, and significant heterogeneity in patient populations and treatment protocols.

## Conclusion

AT appears to offer a survival benefit following resection for DC, although this conclusion is drawn with caution due to the significant limitations of the existing literature, most notably the lack of RCTs specifically designed for DC. Both chemotherapy alone and chemo-radiotherapy show benefits in survival, with the addition of radiotherapy demonstrating a significant improvement in DFS. However, the optimal adjuvant regimen for DC remains unclear. Although capecitabine is currently recommended as the gold standard for AT of cholangiocarcinoma, this meta-analysis suggests that, specifically in patients with DC, it is premature to endorse any specific regimen over another. Due to the considerable heterogeneity in the available data and the challenges in interpreting the efficacy of different therapeutic strategies, critical appraisal of existing retrospective data, such as presented in this meta-analysis, may help inform current clinical decision-making and highlight emerging practice patterns, although these should be interpreted with caution. Future RCTs specifically designed for patients with DC are urgently needed.

## Supplementary Information

Below is the link to the electronic supplementary material.

Supplementary file 1 (DOCX 446 KB)
