# Supplementary material for: Does adjuvant therapy improve survival in patients undergoing pancreaticoduodenectomy for distal cholangiocarcinoma? A systematic review, meta-analysis and meta-regression
Source: Updates Surg. 2025 Sep 1;78(1):275–86. doi: 10.1007/s13304-025-02383-y (PMC12909366; doi:10.1007/s13304-025-02383-y)
Supplement: Supplementary file 1 — (DOCX 446 KB) [file 13304_2025_2383_MOESM1_ESM.docx]

| Outcome of interest | No. | Hazard Ratio (95%CI) | P-value | I^2^ (%) | Egger^ | Begg^ |
| --- | --- | --- | --- | --- | --- | --- |
| *OS* | 20 | 0.80 (0.73; 0.88) | ***<0.001*** | 98.0 | 0.891 | 0.770 |
| *DFS* | 12 | 0.84 (0.74; 0.96) | ***0.012*** | 96.0 | 0.927 | 0.534 |

**Table 1.** Meta-analysis results.

Legend: CI = Confidence Interval; ^Egger and Begg’s test for publication bias; OS = Overall Survival; DFS = Disease Free Survival.


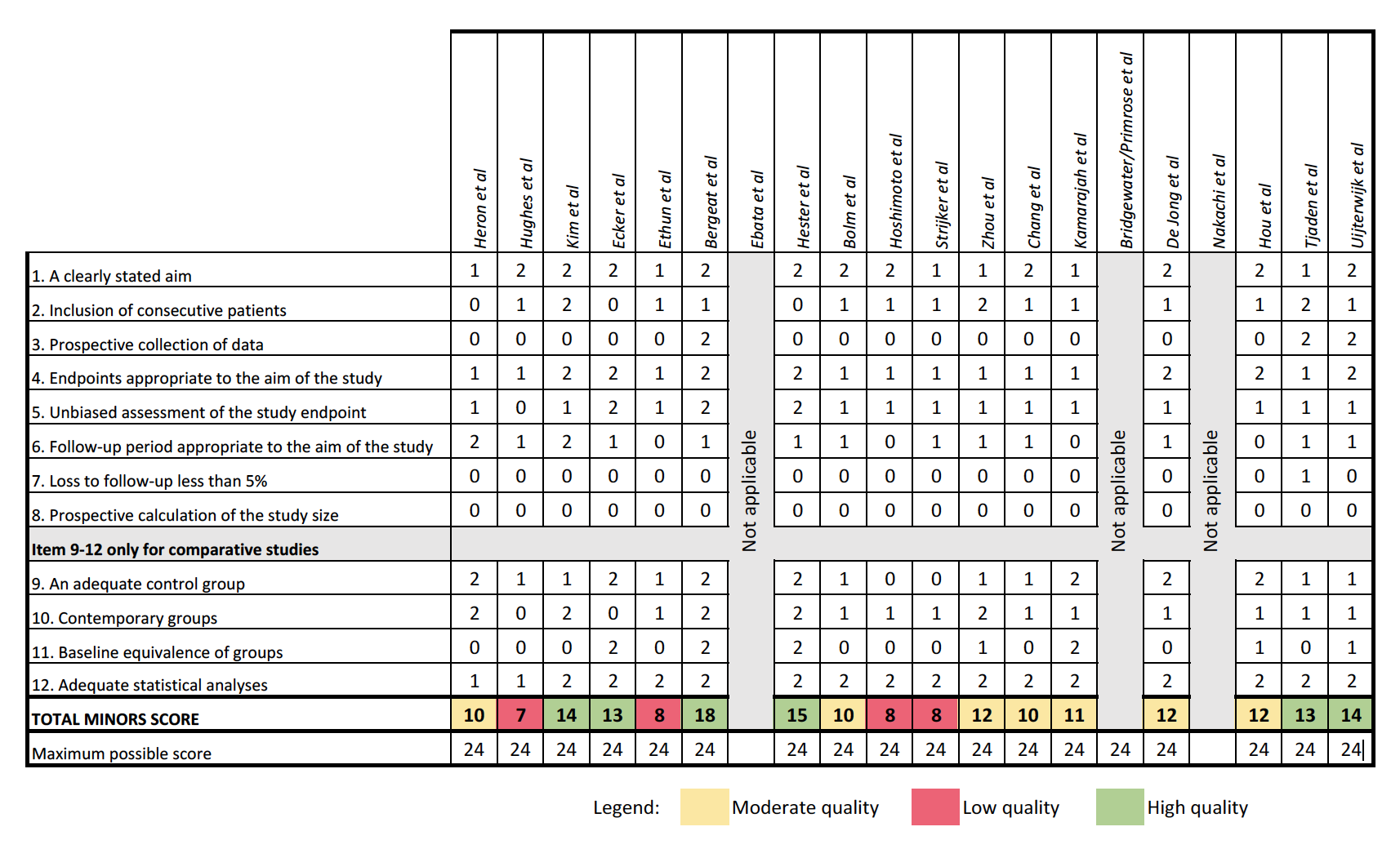


**Figure 1.** MINORs score for retrospective studies


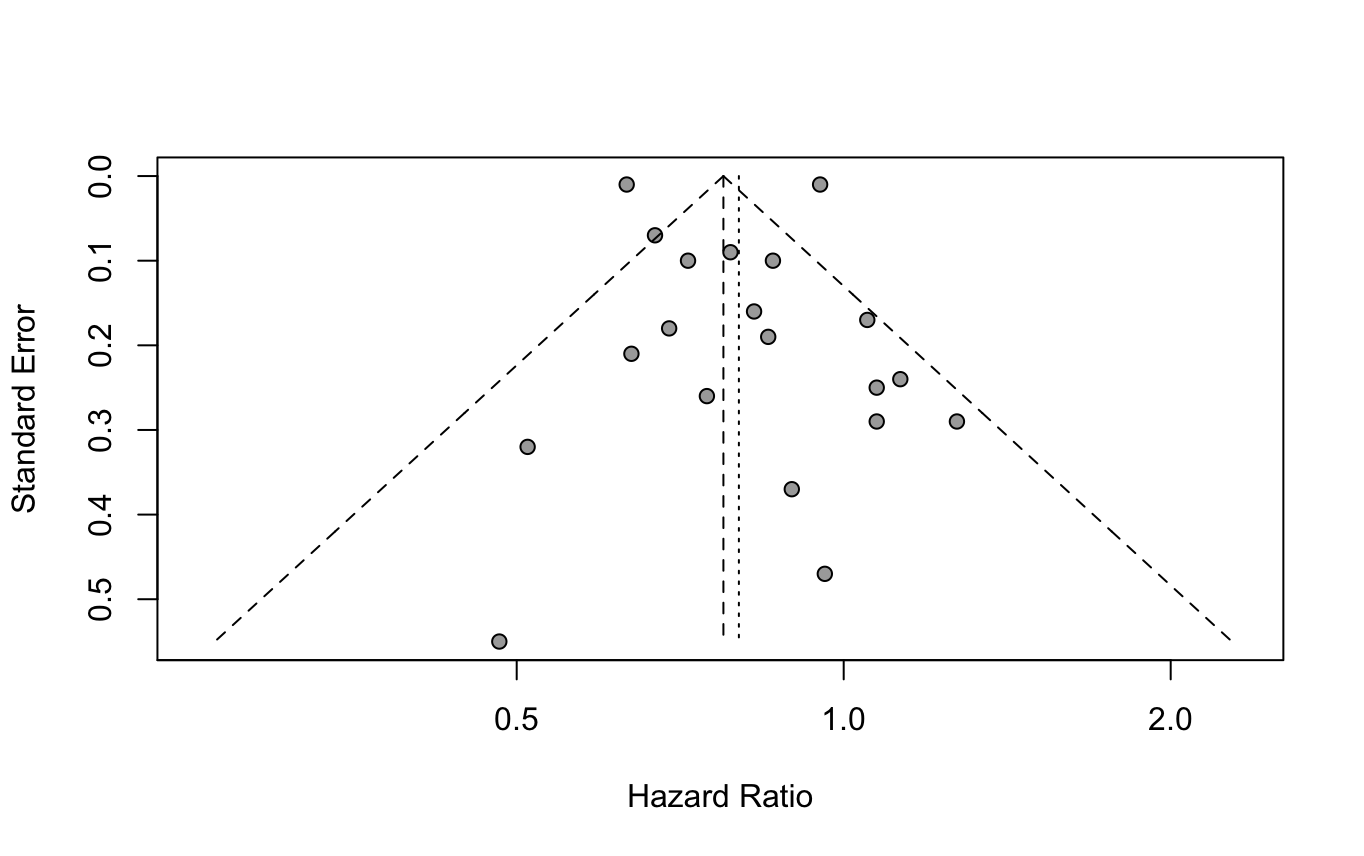


**Figure 2**. Funnel Plot of Overall Survival.


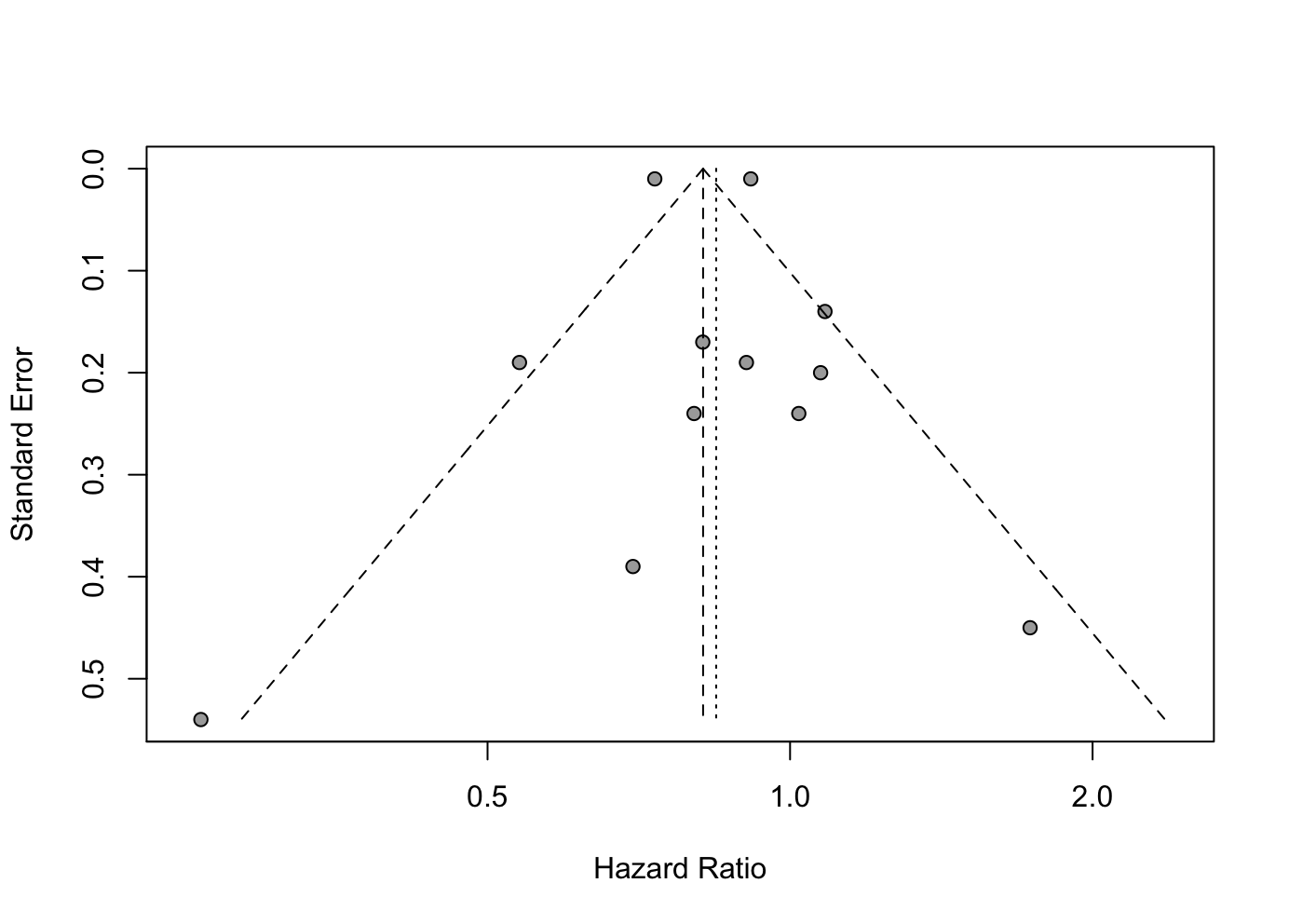


**Figure 3**. Funnel Plot for Disease Free Survival.
